# Patch-Based Recycled Composites: Experimental Investigation and Modeling Techniques on Four-Point Bending and Curved Beam Traction Tests

**DOI:** 10.3390/polym17060757

**Published:** 2025-03-13

**Authors:** Roberto Palazzetti, Lorenzo Calervo, Alessandro Milite, Paolo Bettini

**Affiliations:** 1 Department of Mechanical Engineering, Politecnico di Milano, 20156 Milan, Italy; lorenzo.calervo@polimi.it; 2 Department of Chemistry, Materials and Chemical Engineering “Giulio Natta”, Politecnico di Milano, 20156 Milan, Italy; alessandro.milite@polimi.it; 3 Department of Aerospace Science and Technology, Politecnico di Milano, 20156 Milan, Italy; paolo.bettini@polimi.it

**Keywords:** recycled composites, CFRP, mechanical testing, fracture mechanics, numerical modeling

## Abstract

Composite materials have experienced a significant increase in demand over the past five decades. This growing usage has led to a considerable production of waste, particularly from prepreg scraps, which can account for up to 35% of the purchased material. This paper explores the recycling of prepreg scraps by cutting them into smaller patches and reassembling them into new sheets. The study follows a dual approach: mechanical testing on two different types of samples is presented, along with numerical modeling strategies designed to capture not only the mechanical behavior of the new recycled material but also the failure modes of the samples. The experimental results demonstrate the feasibility of the proposed technique, with samples made from prepreg scraps retaining 85%, 57%, and 78% of the original flexural modulus, strength, and interlaminar strength, respectively. The numerical models not only fit closely to the experimental data but also successfully predict the failure modes of the new material under the two different loading conditions. The primary highlights of this work lie in (i) its innovative approach to recycling prepreg scraps, which is capable of successfully recovering material otherwise sent to landfill; (ii) an ordinated and easy-to-automate recovery process; and (iii) in the modeling strategies of the new material. The study eventually proposes the development of an “equivalent lamina” made of scrap material that can be used in standard lamination processes to manufacture components with load-bearing capabilities.

## 1. Introduction

Highly engineered composite materials have witnessed remarkable growth in popularity and application over the past five decades. Since their introduction more than 50 years ago, these materials have transformed numerous industries due to their exceptional strength-to-weight ratio, lightweight properties, durability, and impact resistance. Today, composites are widely used in sectors such as aerospace, maritime, wind energy, public transportation, construction, sports equipment, and medical devices [1,2], with demand continuing to rise. Projections indicate that by 2030, the use of composite materials in the wind turbine sector alone will increase from 30 to 200 kton. In the pressure vessel industry, demand is expected to reach 180 kton, while advancements in low-cost composite manufacturing could lead to production volumes of up to 100 kton [2]. Along with such rapid growth, end-of-life components and production of waste throughout the manufacturing chain have also increased significantly. Prepreg scraps generated during cutting operations can account for up to 35% of the purchased material [3,4], and much of this waste is currently landfilled, contributing to environmental pollution. The development of sustainable waste management strategies is therefore essential to address rising landfilling costs, meet regulatory requirements, and promote circular economy practices [5].

A variety of mechanical, thermal, and chemical techniques are currently available for the recovery of end-of-life composite materials [6,7,8,9], but this article focuses on managing uncured prepreg scraps, which retain valuable properties suitable for reuse. The objective here is to progress and extend the work of the same authors presented in [10]: two different coupons have been manufactured out of prepreg scrap, tested, and numerically modeled in order to move the recycled material towards a design process for its employment in real world applications. The core of the work is to propose what is here called “equivalent lamina”, a similar concept to that proposed by other researchers [3,11,12,13]. However, among the existing literature, our proposed approach is unique in terms of ordinate orientation and low thickness of the equivalent lamina: in [12,13], for example, the patches are distributed in a plane, and only very thick plates are manufactured, while in [3], dealing with what they call reused scrap roll form (RSRF), the new sheets have patches randomly oriented, significantly affecting the final performance of the laminae.

The use of prepreg scraps has been also explored by other researchers [14,15,16], who have tested recycled rolls and artifacts by randomly depositing and curing patches of various sizes and aspect ratios, demonstrating that recycled materials maintain promising mechanical properties. This new class of material is referred to as discontinuous fiber composites (DFCs), and the literature agrees on the fact that structures built with a regular internal layout exhibit improved strength and stiffness, as they help reduce voids and resin pockets, despite higher manufacturing costs. Despite being out of the scope of this present work, it is worth noticing the results of Nakagawa et al. [17], who showed that DFCs, other than being a suitable response to the necessity of reducing the footprint of the composite industry, are not negatively affected by the aging of the starting material. In fact, such new material can exhibit improved properties with aging time, representing a useful destination not only for prepreg scraps but also for out-of-spec rolls.

Alongside experimental tests, numerical simulations are essential for modeling discontinuous fiber composites, facilitating parametric studies and component design. The literature provides numerous studies on this topic, each proposing distinct methods to capture the complex structure of recycled composites. Nakagawa et al. [17], for example, developed an algorithm to create numerical models of inhomogeneous mesostructures characterized by the random orientation and distribution of patches. Similar methodologies have been adopted also by other researchers [13,18,19]. Nachtane et al. [20], instead, modeled a representative volume element (RVE) of recycled composite material, characterized by highly disordered patterns resulting from uncontrolled deposition and local flow effects during molding. They leveraged a mathematical algorithm developed by Harper et al. [21] to assess patch location, orientation, intersections, and through-thickness distribution, thereby evaluating the global mechanical performance of the heterogeneous composite. A comparable strategy was employed by Belliveau et al. [22], who implemented an algorithm with a uniform statistical distribution for patches positioning and orientation. This approach enabled the setup of a finite element model for discontinuous long fiber (DLF) specimens, facilitating predictions for standard tensile test outcomes. In contrast, Martulli et al. [23] adopted a different approach by bypassing explicit mesostructure modeling, generating an equivalent laminate using stochastic orientation tensors, demonstrating significant potential for scaling applications to larger components. Kravchenko et al. [24] developed a finite element model to predict the tensile properties of composites made from preformed recycled laminae. In their design, prepreg patches were unidirectionally aligned within each layer but staggered across multiple layers. Through subsequent homogenization, macroscopic properties were derived, and the model demonstrated high accuracy in reproducing experimental properties and failure modes, providing valuable insights into the structure–property relationship.

The primary objective of this paper is to develop a numerical model capable of simulating the behavior of the patch-based specimens proposed here, assembled according to a well-defined and organized architecture. A deterministic approach is adopted to accurately replicate the specimen geometry and behavior under two different types of load. Therefore two types of samples have been tested with the double purpose of (i) assessing their mechanical properties and (ii) defining the basis for numerical modeling. The novelty the authors aim to bring lies not only on the new, arranged, thin equivalent lamina but also on the two modeling approaches here presented, which can be used to model real samples with ease by users and designers, depending on the loading conditions and the expected failure modes.

This paper is structured as follows: Section 2 describes the architecture and the manufacturing of the recycled samples, as well as their numerical modeling; Section 3 reports both mechanical and numerical results, alongside with a critical discussion of the findings; finally, Section 4 presents the key conclusions of the study.

## 2. Materials and Methods

In this section, detailed methodologies for sample preparation, testing, and modeling are presented.

### 2.1. Prepreg Patches and Architecture

The material used to manufacture the samples is the HEMT-3 CC601E (purchased by Fibertech, Via Oberdan 334, 21050, Marnate, Italy), a carbon fiber woven 2 × 2 twill weave impregnated with epoxy resin. It has a nominal density of 1.58 g/cm^3^ and a cured lamina thickness of 0.39 mm. Squared patches were cut from the prepreg roll using the Lectra Vector VT-TT-FX-72 (Lectra, Atlanta, GA, USA) automatic cutting plotter. The choice of the patch size plays a crucial role in ensuring a high recycling rate of scraps while maintaining excellent mechanical properties. Aware of this requirement, this study initially opted for patches measuring 50 × 50 mm^2^. This size was chosen as it represents a good compromise between maximizing material recovery (which favors smaller patches) and minimizing the cost, in terms of time and resources, of assembling the new lamina (which benefits from larger patches). Patches were assembled in the so-called “equivalent lamina”, made of two sub-layers:

- the first one is created by placing each patch side by side with no gaps between them until the whole surface is covered;
- the second one is laid on top of the first, shifted by half the size of the patch in both plane directions, ensuring no gaps between patches.

As already detailed in [10], the overlap between patches has been designed to be sufficiently long to prevent it from becoming the weakest point when the lamina is subjected to tensile forces.

This configuration mimics a conventional prepreg sheet: it is used to cut out the shapes required for laminating the desired components. These equivalent laminae can be rolled up and stored in cells to be later used for lamination just like any conventional sheet.

Before diving into the specifics of the patch-based material, it is worth mentioning that an experimental campaign has been conducted on the original reference material to assess its properties: the results are collected in Table 1.

### 2.2. Sample Preparation and Testing

In this work, two kinds of tests are performed: 4-Point Bending (4PB) and curved beam traction tests, and therefore, two different sample geometries have been manufactured. Both samples have been laminated by stacking layers of equivalent lamina until the desired thickness was reached. For the 4PB samples, a flat panel has been manufactured and cured according to the specification provided by the manufacturer; samples have then been cut out using a diamond-blade circular saw.

**Figure 1:**
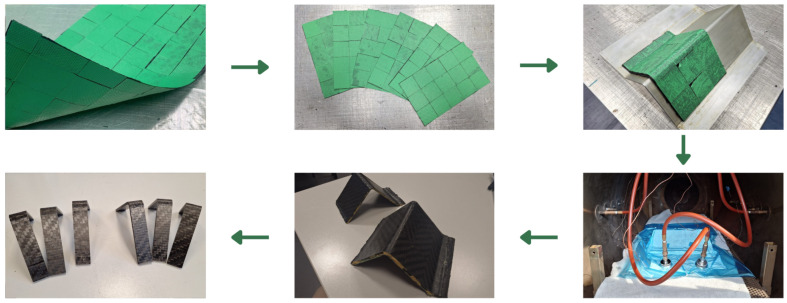
Curved beam sample manufacturing.

Curved beam samples were manufactured following the process shown in Figure 1.

Starting from the recycled equivalent lamina, six sheets were cut out, laminated on the mold, and cured in the autoclave. Reference specimens made with continuous fibers have also been manufactured on the same mold at the same time.

**Figure 2:**
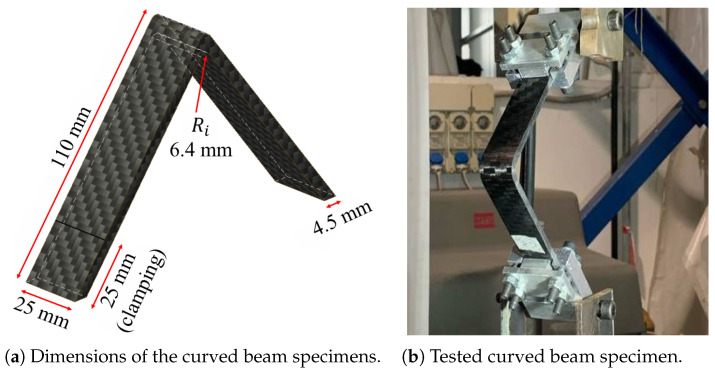
Curved beam specimens and testing apparatus.

Specimens subjected to 4PB tests measured 155 mm in length, 13 mm in width, and 4 mm in thickness, following the recommendation provided in the International Standard [25]. Curved beam specimens, tested under traction conditions, have the dimensions illustrated in Figure 2a, following the recommendations presented in [26]. Both mechanical tests were performed using an MTS 858 MINI BIONIC (MTS Systems Corporation, 14000 Technology Drive, Eden Prairie, MN 55344-2290, USA) testing machine, equipped with an MTS load cell model MTS 4501058 with a maximum load capacity of 100 kN. Moreover, the 4PB tests utilized the deflectometer MTS 632.06H-30 (MTS Systems Corporation, 14000 Technology Drive, Eden Prairie, MN 55344-2290, USA) opt 003&005, following Procedure B of [25]. A support span of 128 mm and a load span of 64 mm were used, with a crosshead speed of 5 mm/min.

Curved beam traction tests required the apparatus shown in Figure 2b, proposed in [26,27]. The specimen tips were clamped using a pair of hinges held by the grips of the tensile testing machine. These hinges were specifically designed to allow free rotational movement of the specimen around the transverse axis.

### 2.3. Modeling

Numerical simulations of the tests conducted on patch-based samples were developed and executed using the commercial software Abaqus 2024®. Two distinct modeling approaches were employed for the two types of samples:

- 4PB sample models are patch-based, and therefore, they include each patch they are actually made of;
- curved beam sample models are equivalent-lamina based, and therefore, each single layer is modeled as an individual equivalent lamina.

Both models share a 2D approach in order to save computational time. The next subsections illustrate the two different approaches in detail.

#### 2.3.1. 4PB Specimen Modeling

**Figure 3:**
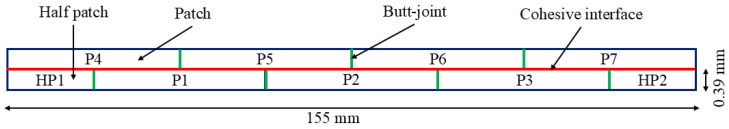
Pattern and numerical model of the first layer of 4PB samples (not in scale).

As mentioned above, a deterministic approach was followed to build the model for the 4PB samples, by modeling each patch individually: Figure 3 outlines the pattern of the first two sub-layers (i.e., the equivalent lamina).

As a result of the alternating pattern, half patches of 25 mm in length arise at the two ends after the desired cutting layout. The same pattern was consistently repeated until the desired thickness was achieved.

**Figure 4:**
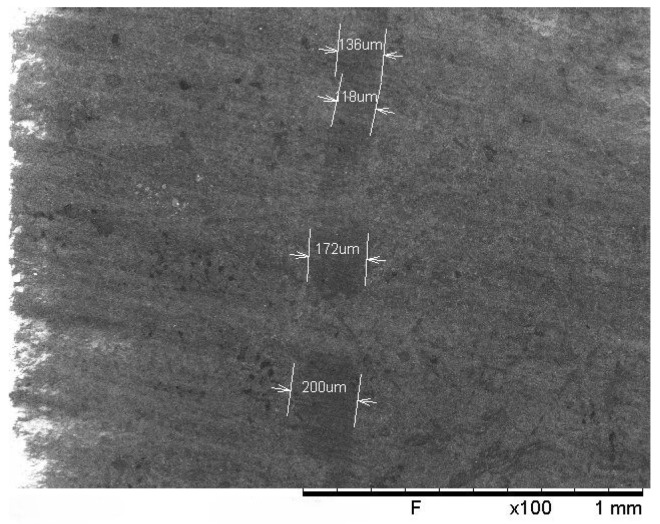
SEM image of butt-joints from the top surface of a specimen.

The main aspects of the model pertain to the interaction between patches. Two different approaches have been followed:

- the first approach relates the interaction between patches from consecutive layers that are in contact on the long side. This interaction was modeled as a cohesive interface, referred to as the “Cohesive interface” in Figure 3, the properties of which were determined through an experimental campaign on the original material mentioned earlier: Double cantilever beam (DCB) and 4-point end notched flexure (4ENF) tests were conducted to determine the parameters for defining the interface properties. While fracture toughness values were directly obtained from experiments (Table 1), the penalty stiffness and the normal cohesive strength were set to 40,000 MPa/mm and 27 MPa, respectively, as a result of the fitting procedure;
- the second approach addresses the interaction between patches within the same layer, in contact along the short edge. In this scenario, the contact area is in reality a resin pocket, as illustrated in Figure 4.

This strip of resin is due to the manual deposition of the patch and to the discontinuity between them, which is inevitably filled with resin during the curing process. Such areas, referred to as “Butt-joints” in Figure 3, were modeled with 1D non-linear spring connectors to simulate the resin’s brittle behavior when subjected to tension and with a non-penetration interaction to avoid the superposition of nodes under compressive load. The SEM images defined the extension of the butt-joint ($l_{i}\approx 200$ μm), and under the hypothesis of 2% elongation at break ($\epsilon_{max}^{r}$), using the resin properties listed in the material’s data-sheet, both the spring’s maximum displacement ($s_{max}$) and the stiffness per unit width ($D_{11}$) were completely defined:

$$\begin{array}{cc} s_{max}= & \epsilon_{max}^{r}\cdot l_{i}=0.004\;\mathrm{mm}\\ D_{11}=\frac{F_{max}^{s}}{s_{max}}= & \frac{E^{r}\cdot \epsilon_{max}^{r}\cdot h_{p}}{s_{max}}=5850\;N/\mathrm{mm} \end{array}$$

where $F_{max}^{s}$ is the maximum force per unit width experienced by the spring before failure, $E^{r}$ is the resin’s Young’s modulus equal to 3 GPa, and $h_{p}$ is the thickness of the patch equal to 0.39 mm.

Eventually, the model also incorporated the Hashin failure criterion, available in the Abaqus® environment, taking into account the strength of the reference material reported in Table 1. Patches were meshed using 0.1 × 0.1 mm^2^ CPS4R elements, providing good representation of the tractions in the fracture process zone of the cohesive interfaces [28] and ensuring that at least four elements are present through the thickness of each patch, achieving a well-described bending behavior.

#### 2.3.2. Curved Beam Specimen Modeling

**Figure 5:**
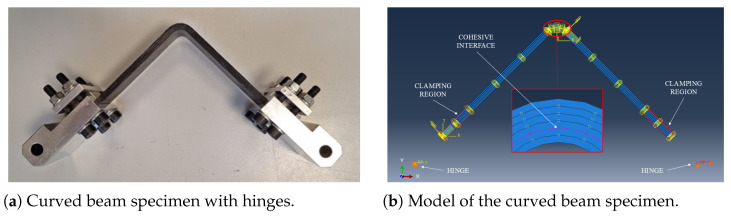
Curved beam real sample and numerical model.

Modeling patch-based components for real applications appears unfeasible mostly due to the very long time needed to build the model; therefore, a different approach has been followed for the curved beam samples, modeled with their equivalent lamina. Each equivalent layer has been modeled as a whole, using properties (such as stiffness and strength) obtained from 4PB tests run on patch-based samples. In this way, layers of 0.78 mm have been assembled to replicate the shape of the curved beam samples. This approach simplifies both numerical modeling and analysis, ensuring consistent mechanical behavior. Once the equivalent lamina is modeled, several layers were stacked to reach the desired thickness. Again, surface-to-surface contact interactions between consecutive layers were addressed with cohesive interfaces (with the same mechanical properties used for the 4PB model) to mimic the adhesive effect of the resin between two equivalent laminae, while springs were unnecessary as the model does not include butt-joint connections. As for the mesh, two different dimensions were adopted: the two arms have been meshed with 0.050 × 0.1 mm^2^ CPS4R elements while the corned region with 0.025 × 0.050 mm^2^ CPS4R elements in order to track delaminations. Figure 5 shows a specimen ready to be tested with its corresponding numerical model. This approach offers significant savings in model construction. Although the number of elements remains unchanged, the patch-based model requires a much larger number of parts (one per patch), especially for long samples. Consequently, it also demands a greater number of cohesive interactions and the use of springs, the latter of which is not needed in the equivalent lamina model.

## 3. Results and Discussion

### 3.1. Experiments

In this section, the experimental and numerical results are presented and thoroughly discussed. The experiments have three main purposes:

- evaluating mechanical properties of the patch-based material to assess its potential to be used for load-bearing components;
- building up the necessary data set for numerical modeling;
- assessing the patch-based laminate properties under different testing configurations.

**Figure 6:**
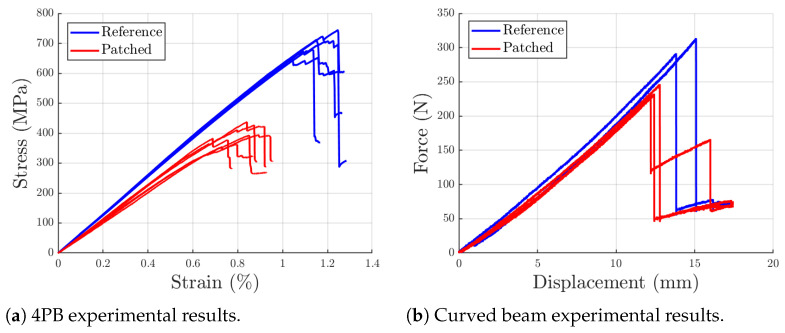
Comparison of reference specimen and patch-based specimen experimental results.

The results shown in Figure 6 clearly show a good retention of mechanical properties for the patch-based laminates, and such retention is maintained similarly across the two different types of tests.

Experimental stress–strain curves from 4PB tests on both standard reference and patch-based materials are shown in Figure 6a. The patch-based specimens exhibit an average Young’s modulus of 54.7 GPa (with a coefficient of variation (COV) of 5.40%) and a strength of 400 MPa (COV equal to 6.98%), corresponding to 85% and 57% retention, respectively, compared to the reference material.

**Figure 7:**
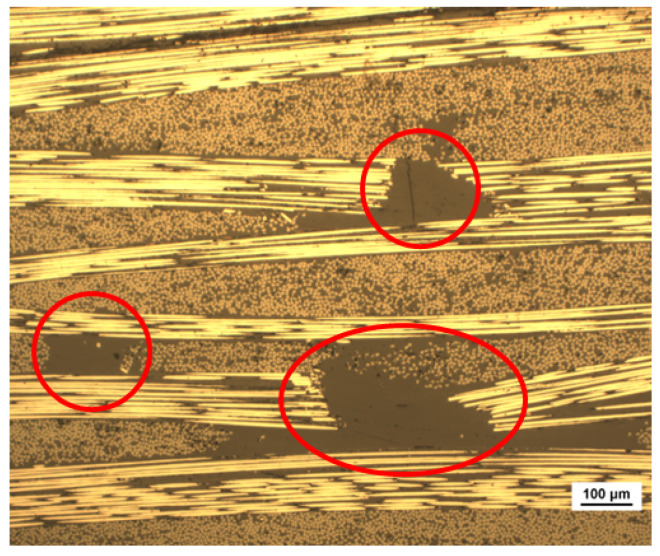
Side view of a patched sample, with resin pockets highlighted in the red circles.

A side view taken from an optical microscope of a 4PB sample is shown in Figure 7.

Microscopic analysis clearly reveals resin pockets at the butt joints of the patches between 200 and 500 microns in length, as indicated by the image scale. These discontinuities are an intrinsic and unavoidable characteristic of patched laminates and contribute to their lower strength compared to long-fiber counterparts. However, it is worth noting that despite the manual lay-up process, curve dispersion is minimal, and the resin-rich areas remain relatively small. This suggests that once the process is automated using a robotic pick-and-place system, patch positioning will improve and discontinuities will be further reduced.

A comparison between the results presented here and previous studies on bending-tested patch-based laminates is provided in Table 2.

It is noteworthy that other studies in the literature propose a random orientation of the patches, which results in a cheaper new laminate. However, the organized architecture introduced here allows for much better property retention compared to the original long-fiber material. The only study that reports better properties, albeit only in terms of strength, is that of De Souza et al. [14], where, however, much larger patches are used, reducing the recycling potential of the technique compared to the 50 × 50 mm^2^ patches presented here.

Regarding curved-beam samples, the authors could not find any prior work testing similar samples made of short-fiber composites, thus making the comparison impossible. As shown in Figure 6b, patch-based specimens achieved an average maximum force of 237 N (COV equal to 3.5%) compared to the 303 N (COV equal to 5.2%) achieved by the reference samples, thus showing a 21.7% reduction in peak load. Moreover, stiffness can be evaluated comparing the slopes of the force-displacement diagrams: the patch-based specimens showed a stiffness of 19.8 N/mm (COV of 0.9%) while their counterparts a stiffness of 21.2 N/mm (COV of 1.5%), which translates into a 6.3% reduction.

Under 4PB conditions, the patch-based mesostructure significantly influences the final strength of the specimens. Resin-rich regions between patches act as weak spots, facilitating crack propagation and leading to earlier failure. Conversely, stiffness is only weakly affected by the discontinuous pattern. Since the fiber length remains well above the characteristic length (typically less than 0.5 mm for carbon fiber reinforced epoxies [29,30], the reinforcing effect is fully realized. The small reduction in stiffness may be attributed to a slightly higher resin volume fraction in the patch-based specimens.

Similarly, the patch-based mesostructure determined an effect on the out of plane interlaminar strength of curved beam specimens, whose value $\sigma_{r_{max}}$ can be computed by means of an elementary beam theory, as proposed in [27]:

(1) $$\sigma_{r_{max}}=\frac{3P(Lcos(\omega )-(\Delta +0.5t)sin(\omega ))}{2bt{\left(r_{i}\left(r_{i}+t\right)\right)}^{1/2}}$$

where P is the maximum load; L is the arm length (distance from the point at which the load is applied and the beginning of the corner region); ω is the angle between the axis of the hinges and the vertical reference; Δ is the distance between the hinges and the specimen’s surface; t and b are specimen’s thickness and width, respectively; and $r_{i}$ is the internal radius of the sample. This approach results in an interlaminar strength of 23.1 MPa (COV of 3.5%) and 29.5 MPa (COV of 2.5%) for the patch-based and the reference material, respectively, highlighting a 21.7% retention, the same as the peak load.

**Figure 8:**
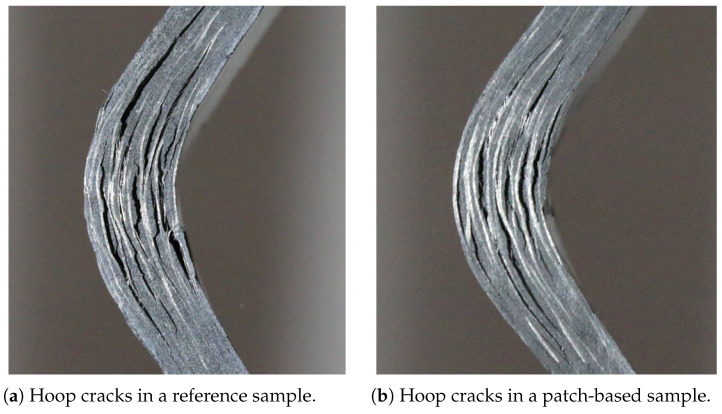
Hoop cracks in tested samples.

The experimental testing on curved beam specimens also showed the same failure modes for both architectures, as shown in Figure 8, indicating that the complex mesostructure of the patch-based specimens does not significantly influence stress distribution. This suggests that the cause of lower strength is a stress intensification in correspondence with the edge of the patches, together with a bending–twisting coupling, which causes a premature insurance of the delaminations, as already observed in the literature [14].

Overall, for both 4PB and curved beam specimens, the patch-based material has shown valuable mechanical properties: this not only validates the recovery approach adopted in this work but also opens the possibility of manufacturing load-bearing components out of the equivalent lamina made of recovered prepreg patches.

### 3.2. Numerical Simulations

**Figure 9:**
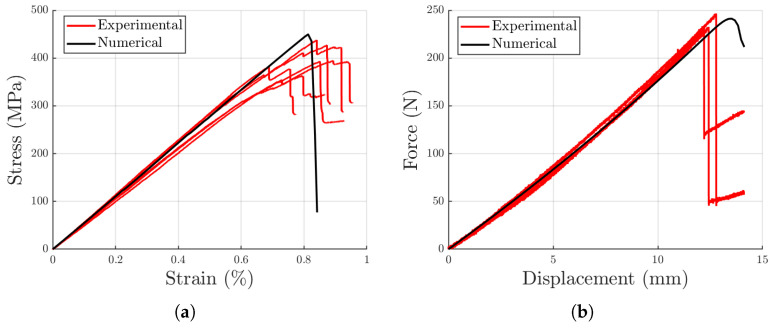
Comparison between experimental and numerical results for 4PB and curved beam traction tests. (**a**) Experimental and numerical 4PB curves. (**b**) Experimental and numerical curved beam traction curves.

After assessing the mechanical properties of the patch-based laminates, numerical models for both sample types were developed to establish a design method for this recycled material. The models, constructed following the approaches described in Section 2.3.1 and Section 2.3.2, demonstrated excellent agreement with the experimental results, as shown in Figure 9.

Both models accurately follow the experimental trends, both in terms of loading path and of maximum load, demonstrating their utility as tools for designers for future study and development of the recycling method proposed in this work. Specifically, simulations predicted a strength of 450 MPa and a stiffness of 55.3 GPa, showing discrepancies of 11% and 1.2%, respectively, when compared to experimental results from patch-based samples. For the curved beam samples, the numerical model calculated a maximum force of 241.2 N and a stiffness of 18.6 N/mm, resulting in differences of 1.7% and 6.4%, respectively, compared to the experimental data.

**Figure 10:**
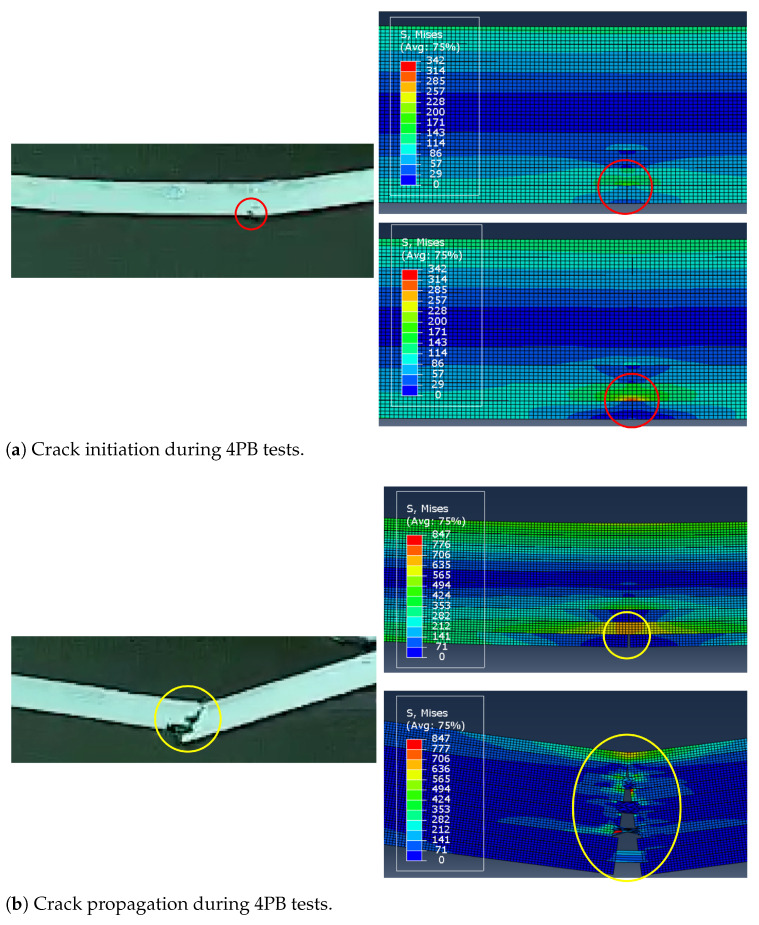
Comparison between failure of 4PB specimens observed through high-speed imaging (**left**) and resulting from the finite element simulations (**right**).

Upon closer examination, the models not only predict strength and stiffness of the two samples but also capture the failure modes observed in the experiments. The 4PB tests were recorded using a Phantom VEO-E 310L high-speed camera at a rate of 140 kHz. The video showed that during the loading phase of the test, the first failure occurred at the butt-joint between patches on the tension side of the specimen (Figure 10a, left), without affecting the initial linear trend of the stress–strain curves. However, this initial detachment marked the initiation of crack propagation. Once the maximum load was reached, the samples failed abruptly due to fiber breakage in the subsequent plies (Figure 10b, left). A more detailed investigation of the samples’ failure during the experiments is provided in [10]. Numerical simulations followed the same trend. Figure 10a (right) illustrates the moments before and after the butt-joint failure, showing how the load is transferred from the lowest to the second-last layer once the butt-joint (i.e., the spring) fails. Figure 10b (right) shows the fiber breakage occurring at the peak load, just as the test ends.

From the numerical simulations, and particularly in Figure 10a (right), it can be observed that once the spring breaks, i.e., when the butt-joint fails, the load is transferred to the upper layer. This induces a sudden and sharp stress concentration on the patch, which ultimately causes the component to fail. As shown in the simulations presented here, and particularly in Figure 10b (right), just before failure, the stress is around two times higher than in the neighboring areas at the same distance from the neutral axis. It can be concluded that resin-rich areas are not directly responsible for the reduced resistance of the samples, but they contribute to stress concentration within the lamina, which eventually leads to specimen failure.

**Figure 11:**
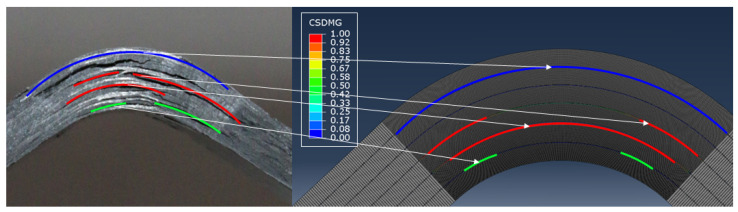
Crack development in the corner region: experimental and numerical pattern comparison.

Curved beam traction tests were not recorded with a camera, but the failure modes are clearly identifiable by examining the tested specimens. Specifically, these specimens exhibit hoop cracks—delaminations—that developed suddenly at the peak load of the test. Once the delaminations occurred, the load dropped sharply, and this behavior was accurately captured by the simulations. Additionally, by examining the detached elements in the numerical model after the tests, delaminations similar to those observed in the real specimens can be identified. Figure 11 highlights the delaminations in the corner region, providing a comparison between numerical and experimental outcomes.

Despite the delaminations occurring simultaneously at the interfaces, the numerical models were able to track their progression and the weakening of the interfaces as the load increased. According to our model, the first interfaces to fail are those located at the center of the specimens, followed by those on the lower side, i.e., the side under tension. Fully delaminated layers are highlighted with red lines in Figure 11 (right). The interfaces highlighted in blue, on the other hand, are those farthest from the delamination, which did not occur in the upper part of the real samples. In the lower region, the experiments showed some minor delaminations, while the model indicated areas with a damage index around 40%, suggesting that delamination is likely to occur. Finally, it is important to note that the model fails to predict delaminations occurring between the equivalent lamina, i.e., due to the detachment of the two sub-layers.

## 4. Conclusions

This paper addresses the urgent need to reduce the environmental impact of the composite industry, which currently sends a significant portion of purchased material—still in good condition—to landfills due to scraps and end-of-roll waste. The proposed recycling method differs from other techniques (such as thermomechanical fiber reclamation, pyrolysis, or other thermal or chemical processes) because it addresses original, unprocessed material that is still in good condition. The focus is not on end-of-life components but on preventing the landfilling of material that remains in its original, usable state. The main highlight of the work is the development of the so-called “equivalent lamina”, which is built by assembling prepreg scraps cut into smaller and regular shapes: the new lamina can be rolled, stored, and later used like in any conventional lamination process.

A second highlight of the work is that the new recycled material retains a significant load-bearing capacity, maintaining 85%, 57%, and 78% of the original reference flexural modulus, strength, and interlaminar strength, respectively, consistently under two different load conditions. Furthermore, the results showed minimal variability in the experimental curves and consistency across the two testing methods. A third main result of the work is related to the modeling of the new lamina. Two numerical models have been proposed: a patch-based model, where each patch is individually modeled, and an equivalent lamina-based model, where each equivalent layer is treated as a whole. This latter approach ensures consistent mechanical behavior while saving significant building time. Both models successfully replicated the experimental curves and captured the failure modes under the two loading conditions. It is important to note, however, that the two models are not equivalent, and the choice between them depends on geometry and loading conditions. The patch-based model can capture all possible failure modes, including butt-joint failure, from which a crack may initiate and propagate. However, constructing complex shapes with this approach can be highly time-consuming. Conversely, for components expected to undergo delamination rather than fiber breakage, the equivalent lamina approach provides the same reliability and fidelity while significantly reducing build time. The development of a hybrid model that combines the advantages of both strategies is the next step in our research.

It is also worth mentioning that the authors have experimentally tested and numerically modeled different patch sizes (i.e., 34 × 34 and 60 × 20), and the results are consistent with those presented here.

The results presented aim to lay the foundation for a new strategy to recycle valuable materials that are currently destined for landfills. When working with recycled materials, designers require accurate information on material properties and design constraints, which is the primary motivation for this work. The authors believe that a recycled composite material that retains mechanical properties similar to those of the original material and that can be processed like a conventional prepreg sheet has the potential to be used in a wide range of applications, including load-bearing components.

This paper claims to offer valuable insights for further developing the proposed recycling strategy and to provide solid foundation for creating accurate design strategies that can reliably predict the behavior of these new materials.

## Figures and Tables

**Table 1:** Fibertech HEMT-3 CC601E mechanical properties.

Property	Value
Young Modulus	64.3 GPa
Strength	700 MPa
$G_{I_{C}}$	1.356 kJ/m^2^
$G_{II_{C}}$	2.378 kJ/m^2^

**Table 2:** Existing works on patch-based laminates tested in bending.

	This Work	[14]	[17]	[17]
Material	HEMT-3 CC610E	HexPlyF155 W3T282	T800S/3900	T800S/3900
Platelet size (mm^2^)	2500	15,000	114	285
Bending Strength (MPa)	400 ± 28	371 ± 55	233 ± 60	456 ± 116
Strength retention	57%	44%	8%	15%
Bending Stiffness (GPa)	55 ± 3	55 ± 3	32 ± 4	39 ± 2
Stiffness retention	85%	91%	22%	26%

## Data Availability

The raw data supporting the conclusions of this article will be made available by the authors on request.
